# Optimizing the optical and biological properties of 6-(1*H*-benzimidazole)-2-naphthalenol as a fluorescent probe for the detection of thiophenols: a theoretical study†

**DOI:** 10.1039/d0ra04835f

**Published:** 2020-06-25

**Authors:** Przemysław Krawczyk

**Affiliations:** Nicolaus Copernicus University, Collegium Medicum, Faculty of Pharmacy, Department of Physical Chemistry Kurpińskiego 5 85-950 Bydgoszcz Poland przemekk@cm.umk.pl +48 52 5853679

## Abstract

The study presents the influence of structure modulation by introduction of selected donor and acceptor substituents on the properties of 6-(1*H*-benzimidazole)-2(2,4-dinitrobenzenesulfonate)naphthalene used in thiophenol identification. The presence of –OH and –OR groups enhances the non-linear optics (NLO) response of the marker. The –NO_2_ substituent maximizes the non-linear response and increases the amount of transferred charge and the charge-transfer distance. The introduction of the –OH, –NO_2_ and –CN groups into the marker structure significantly improves the solubility and optical availability. The –NO_2_ group however contributes to mutagenicity and carcinogenicity. The –OH and –OR groups can be successfully used in bioimaging to detect specific molecules containing the –SH group in their structure. At the same time, the –OR group minimizes the energy barrier necessary to break the bond between the chromophore and the linker. The paper also includes a comparison of optical and biological properties of structures before and after identification of thiophenols.

## Abstract 2

The study presents the influence of structure modulation by introduction of selected donor and acceptor substituents on the properties of 6-(1*H*-bezimidazole)-2(2,4-dinitrobenzenesulfonate)naphthalene used in thiophenol identification.

## Graphical abstract

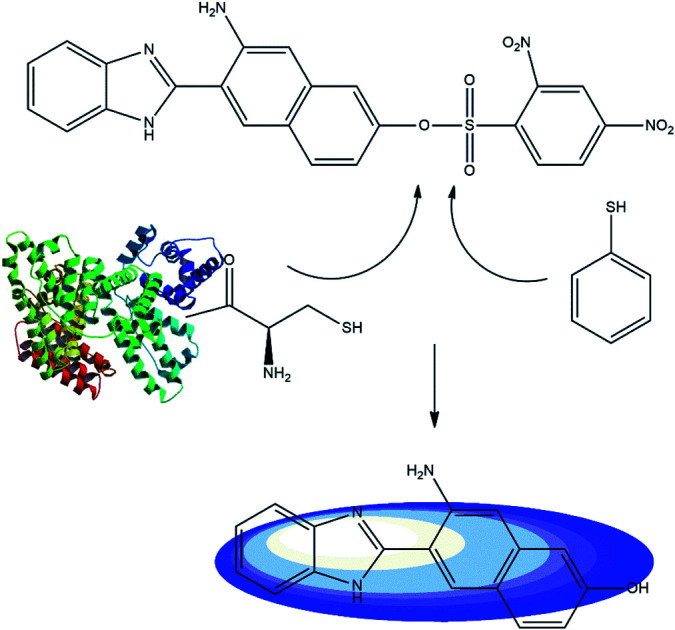

## 1 Introduction

Thiophenols are widely used in the synthesis of many industrial products, primarily polymers, pesticides and pharmaceutical products.^1,2^ Despite their widespread use, they are highly toxic and the average lethal dose (LC_50_) for fish is only 0.01–0.04 mM.^3^ Also, the human body is very exposed to the toxic effects of these substances due to inhalation and rapid absorption through the skin. Exposure of the human body to these compounds results in many serious illnesses, including polyposis, nausea, vomiting, muscle weakness and in special cases even death.^4,5^ Against this background, it is particularly important to search for new solutions for the easy and quick detection of thiophenols, especially in pharmaceuticals and in biological material. In this context, fluorescent probes, which are dyes relatively easy to manufacture with a wide spectrum of Stokes shifts, have become of practical importance. It is easy to modulate the structure of such compounds in order to optimize desirable photophysical features and they can be used in *in vivo* and *in vitro* tests. For detecting thiophenol derivatives, fluorescent probes are designed in such a way that the fluorophore is linked to an appropriate quencher unit to transfer the transformed molecule to another group with no quenching capability or cleaved from the fluorophore permanently. In this way, fluorescence is recovered and the last derivative is responsible for marker fluorescence. An example is 2,4-dinitrophenylsulphonyl (DNS).^6–9^ Many probes tailored for the detection of thiophenols are designed on the basis of the reaction between thiophenols and the sulfamide unit (RNH–SO_2_R′). However, thiophenol detection *via* such probes is relatively long and takes on average 20–30 min.^10–12^ The search for more effective solutions leads to the use of a sulfonyl ester (RO–SO_2_R ′), which significantly reduces the detection time.^13^

**Scheme 1:**
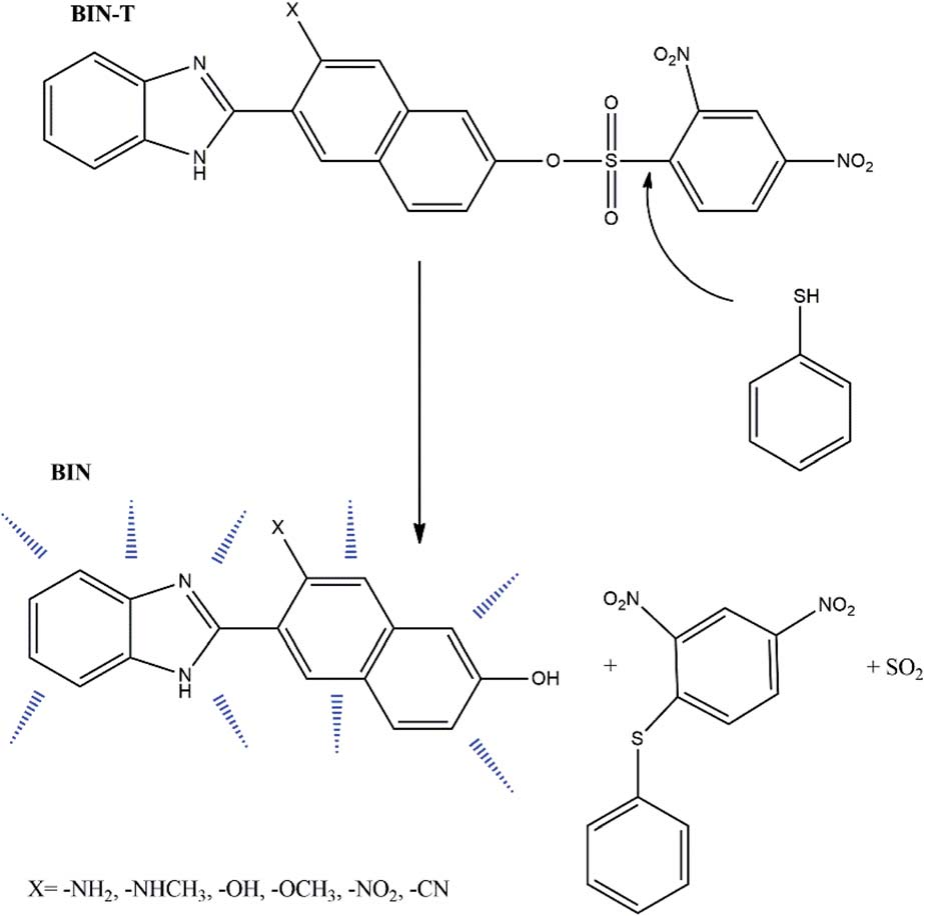
Detection pathway of thiophenol derivatives.^14^

Based on this second strategy of fluorophore construction, the W. Lv^14^ group has synthesized 6-(1*H*-benzimidazole)-2-naphtalenol (BIN) as a satisfactory probe for the detection of thiophenols. It has been shown to have excellent photostability, good biocompatibility and a large Stokes' shift in aqueous solutions. By connecting the BIN to the sulfonyl ester group, the 6-(1*H*-benzimidazole)-2(2,4-dinitrobenzenesulfonate)naphtalene (BIN-T) probe was synthesized. In this construction, BIN is a fluorophore, while the 2,4-dinitrobenzenesulfonyl group is both a thiophenol recognition group and a fluorescence quenching unit (Scheme 1). For the marker constructed in this way, the average detection time of thiophenols was about 10 min.

Due to the possibility of using BIN for the detection of thiophenols in pharmaceutical products and in biological material, it was decided to check which factors enhance the linear and non-linear optical response and improve biological properties. For this purpose, electron-donating and electron-withdrawing substituents have been attached to the naphthalene part. In this way, six derivatives (Scheme 1) with different photochemical and biological properties were obtained. The presented research results allow to answer the question how the probe structure should be modulated to obtain a marker with the desired photochemical properties required for molecular imaging. The study of marker structure modulation by changing substituents has a very important cognitive purpose. On the one hand, it allows achieving the desired optical and biological characteristics. On the other hand, it allows the creation of a fluorescent probe operating in a specific absorption and fluorescence range, depending on the research requirements.

## 2 Synthesis path

**Scheme 2:**
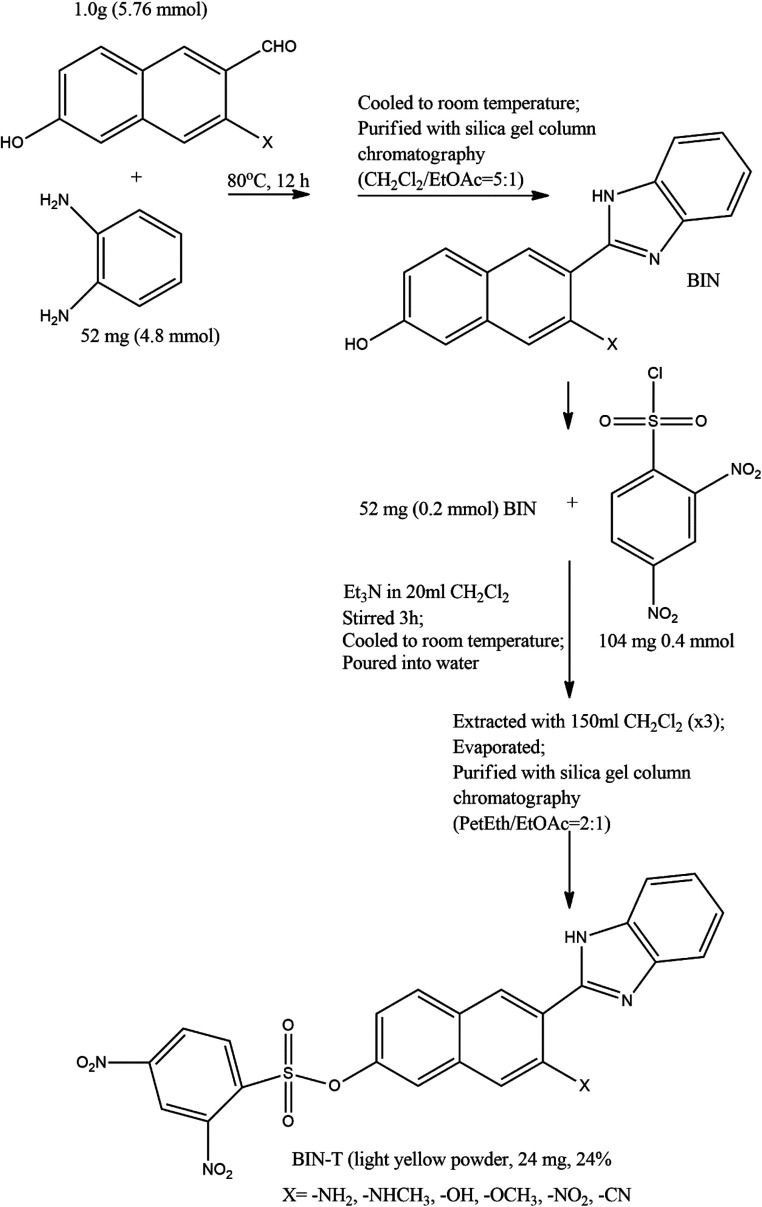
Synthesis of BIN-T probes (see ref. 14 for further details).

The method of synthesis of the tested derivatives is presented in Scheme 2.

## 3 Computational details

All geometrical parameters of investigated molecules in their ground (S_GS_) and excited states (S_CT_) were calculated using density functional theory (DFT) approach implemented in Gaussian 09 program package^15^ with TIGHT threshold option and PBE0/6-311++G(d,p) basis set. In order to verify that all the structures correspond to the minima on the potential energy surface, an analysis of Hessians was performed. The electronic properties were characterized by computations of the vertical absorption and emission spectra, which were obtained using the time-dependent density functional theory (TDDFT/PBE0)^16^ and by including the state-specific (SS) corrected linear response (cLR) approach.^17^ Due to the high compatibility of theoretical and experimental data,^18–21^ all spectroscopic calculations were performed using PBE0 functional.

For the best consideration of the solvent impact on the fluorescence spectra, the ground state should be calculated with non-equilibrium solvation.^22,23^ This was taken into account by including the state-specific (SS) corrected linear response (cLR) approach^24^ to the theoretical calculations. In the SS approach the solvent dynamic polarizations are determined by the difference of the electron densities of the initial and final states.^25–27^

The dipole moments and polarities of the charge-transfer state (S_CT_) were evaluated by numerical differentiation of the excitation energies (*E*) in the presence of an electric field *F* of 0.001 a.u. strength:1
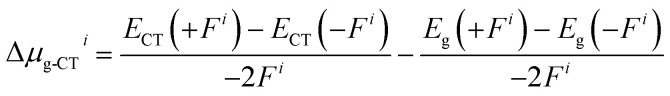
where *i* stands for the Cartesian component of the dipole moment difference and g is ground state (S_g_). The isotropic average polarizability (〈*α*〉), polarizability anisotropy (Δ*α*) and first-order hyperpolarizability (*β*_vec_) were determined based on the Gaussian 09 program and defined as:2
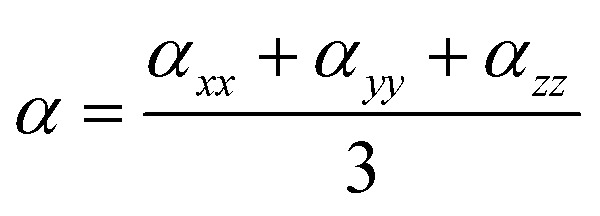
3
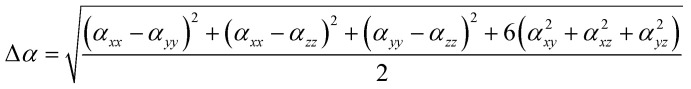
4
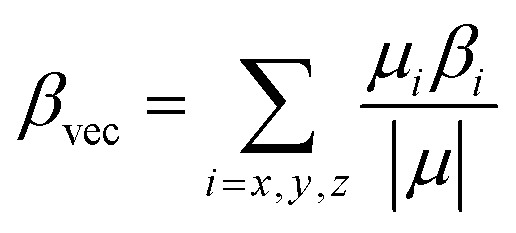
where *β*_*i*_ (*i* = *x*, *y*, *z*) is given by 
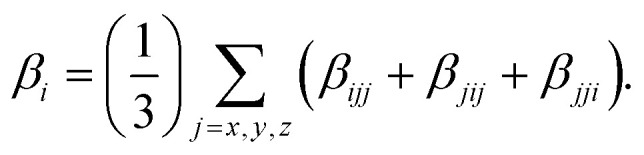


The density differences were obtained at the PBE0/6-311++G(d,p) level and are represented with a contour threshold of 0.02 a.u. In these graphs, the blue (purple) zones indicate density decrease (increase) upon electronic transition. The charge transfer parameters, namely the charge-transfer distance (*D*_CT_) and the amount of transferred charge (*q*_CT_), have been determined following a Le Bahers' procedure.^28^ The solvent effect on the linear and nonlinear optical properties has been taken into account using the Integral Equation Formalism for the Polarizable Continuum Model (IEF-PCM).^29,30^

Experimentally, the two-photon absorption (TPA) can be obtained by the dissipation of the incident light, which for a single beam 2PA experiment is twice the transition rate. In this case, the two-photon cross-section of the degenerate process is written as:^31–33^5
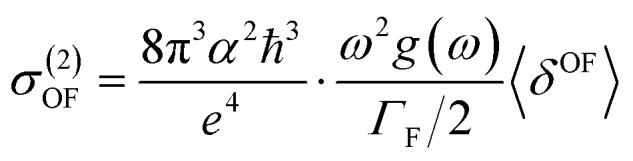
where *α* is a fine structure constant, *ω* is the frequency of absorbed photons (assuming one source of photons), *Γ*_F_ is the broadening of the final state (F) due to its finite lifetime and *g*(*ω*) provides the spectral line profile, which often is assumed to be a *δ*-function and 〈*δ*^OF^〉 is the two-photon transition probability for the transition from the ground state to a final state.

In the case of a molecule absorbing two photons of the same energy in isotropic media, the degenerate 〈*δ*^OF^〉 in an isotropic medium using a linearly polarized laser beam given by:^34^6
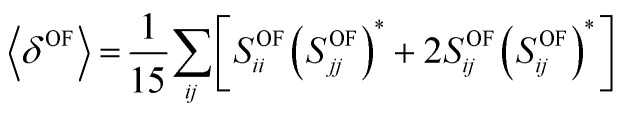


In this equation, *S*^OF^_*ij*_ is the second-order transition moment given by:7
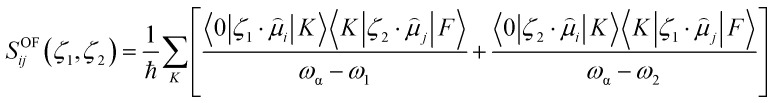
where *ℏω*_1_ + *ℏω*_2_ should satisfy the resonance condition and 
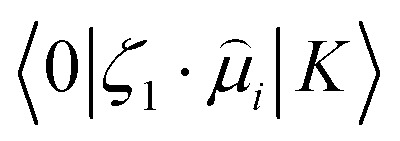
 stands for the transition moment between electronic states |0〉 and |*K*〉, respectively. *ζ* is the vector defining polarization of photons. To describe the two-photon allowed states, the quadratic response functions formalism^35,36^ within the DFT framework was used as implemented in the DALTON 2011 program.^37,38^ Solvent effects were taken into account with the self-consistent reaction field (SCRF) model. All the 2PA calculations were carried out employing the CAM-B3LYP functional and the 6-311++G(d,p) basis set.

The biological activities were simulated using a combination of the 3D/4D QSAR BiS/MC and CoCon algorithms.^39–43^

The binding properties of considered dyes were studied by performing series of AutoDock 4.2 (ref. 44–46) and AutoDock Vina^47^ simulations. For each active complex comprising Human Serum Albuminum (HSA), their crystal structures were taken from PDB ID: 1AO6.^48^ The cubic grid box with the dimensions of 16 Å and a grid spacing of 1 Å was set up in such way that the reactive –NH_2_ groups of lysine and –SH of cysteine were at its centre. In order to identify appropriate binding energy and conformation of compounds, the Lamarckian genetic algorithm was employed. For each atom of the receptor molecule Gasteiger charges were calculated. The investigation of the binding site was performed using a united-atom scoring function. For each amino acid the docking simulations were performed tenfold.

## 4 Results

### 4.1. Physicochemical properties

**Fig. 1:**
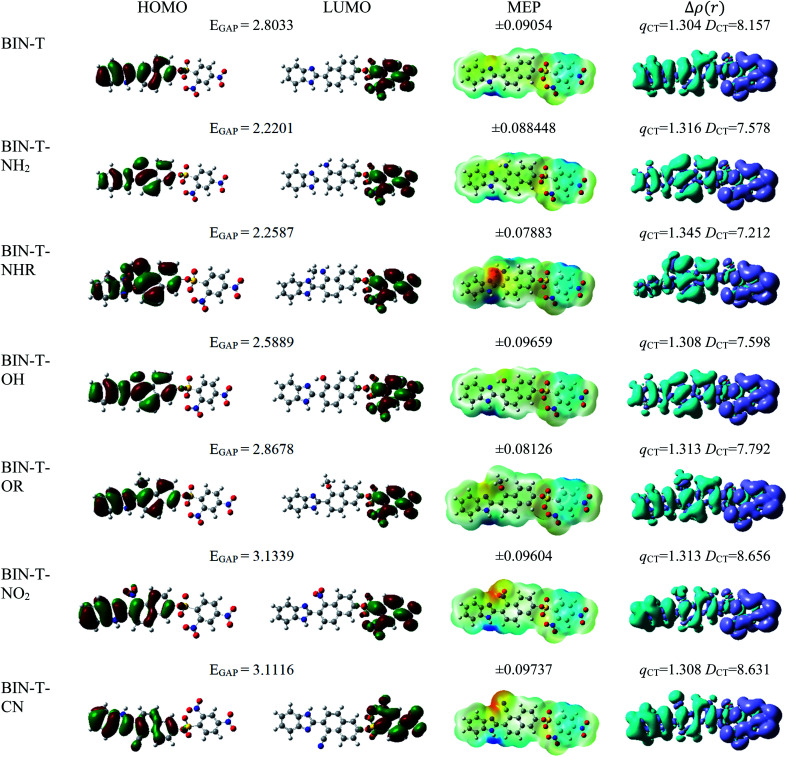
Physicochemical properties of BIN-T derivatives.

**Fig. 2:**
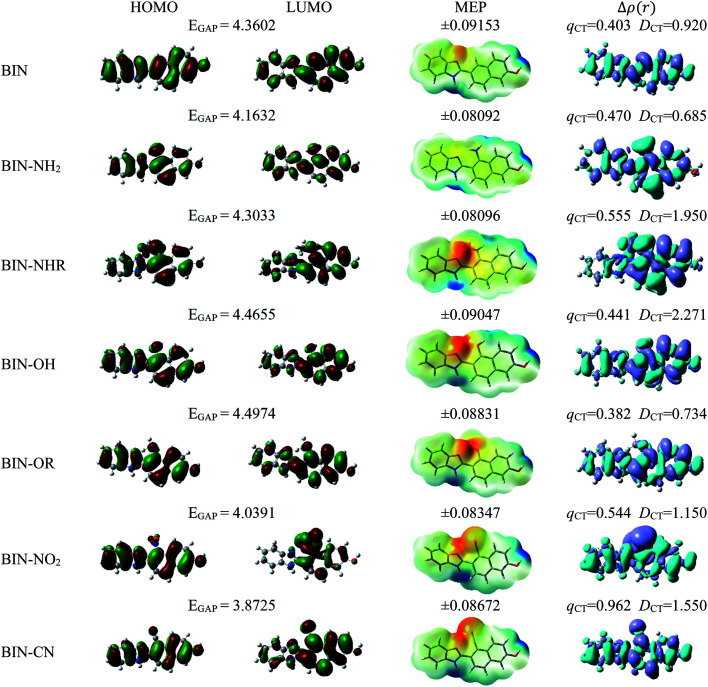
Physicochemical properties of BIN derivatives.

For all investigated molecules, the charge-transfer (CT) excitation corresponds to the HOMO → LUMO transition (Fig. 1 and 2). In the case of the BIN-T probe, HOMO electrons are located on the 6-(1*H*-benzimidazole)-2-naphtalenol part, while LUMO on the 2,4-dinitrobenzenesulfonyl group. The attachment of substituents, as well as their change, does not affect the position of these frontier orbitals. Reaction with thiophenol and obtaining BIN fluorophore significantly changes this distribution. HOMO electrons accumulate throughout the entire molecule, while LUMO mainly accumulates on the naphthalene part. This is particularly apparent for the BIN–NO_2_ derivative. For BIN-T derivatives, there are slight differences in the case of energy separation between HOMO–LUMO orbital (*E*_GAP_) – Table SI1.† Attachment of the –NH_2_ and –NHR substituents reduces *E*_GAP_ by 0.55 eV on average. In turn, the presence of –OH reduces it by 0.2 eV and the presence of –OR brings this value closer to the BIN-T level. The presence of electron-acceptor substituents increases the *E*_GAP_ value by more than 0.3 eV. After thiophenol detection (Table SI2†), the *E*_GAP_ value increases significantly for water Δ*E*^BIN–BIN-T^_GAP_ = 1.5569 eV. In this case, the influence of substituents is slightly different. The presence of –NH_2_ reduces it by 0.2 eV while –NHR does not change this value. The –OH and –OR substituents slightly increase *E*_GAP_ and the electron-acceptor ones decrease it. For both BIN and BIN-T derivatives, no significant effect of solvent presence on the EGAP value is observed. In general, for BIN-T derivatives with increasing polarity of the medium *E*_GAP_ increases (
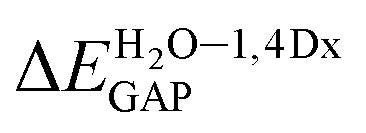
 = 0.1970 eV) and for BIN decreases (
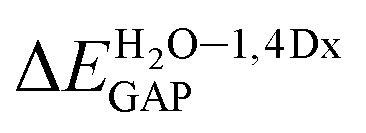
 = 0.0160 eV). In addition, a significant decrease (by more than 1 eV) in chemical hardness (*η*) is observed for BIN-T derivatives. High electronegativity (*χ*) value suggests an easy formation of covalent bonds during various chemical processes.

In order to assess the sites of potential electrophilic and nucleophilic attack, the molecular electrostatic potential (MEP) analysis was performed (Fig. 1 and 2). In the case of BIN-T, the place with the highest electronegativity and thus the most exposed to nucleophilic attack (positive, blue zones) is the nitrogen atom with hydrogen attached in the imidazole part. Benzene in the 2,4-dinitrobenzenesulfonyl part is also a region that may undergo nucleophilic reactions. Substitution with subsequent substituents does not change the location of these places. Only for BIN-T–NH_2_ and BIN-T–NHR positive zones are also observed on the nitrogen atoms of substituents. Sites for electrophilic attack (red and yellow, negative zones) are mainly the second of the nitrogen atoms of the imidazole part and the oxygen atom in the sulfonylester group. The presence of substituents forces the negative zones to shift into the regions of the connected groups. Among the considered derivatives, the highest risk of electrophilic attack is experienced by BIN-T–CN, where the charge on the nitrogen atom of the –CN group is as much as −0.09737 a.u. After the detection of thiophenols and obtaining the BIN structure, the nitrogen atom of the imidazole group is still the place with the highest risk of nucleophilic attack. In addition, an important place for such a reaction is the hydrogen atom of the hydroxyl group. The place for the electrophilic attack remains the second nitrogen atom of the imidazole ring and substitution with subsequent substituents does not change these regions. Similar to BIN-T, negative zones also appear on substituents. It can also be seen that the attachment of –NH_2_ slightly reduces the charge on the nitrogen atom, reducing the risk of an electrophilic reaction.

The studies have been devoted to many reflections on spectral properties, corresponding to the HOMO → LUMO photoexcitation (π–π* transitions). In order to estimate contributions from other orbitals and determine the nature of electronic states, the density variation upon photoexcitation (Δ*ρ*(*r*)) was computed for the first electronic transitions, which is graphically depicted in Fig. 1 and 2. For BIN-T, the figure indicates that the depletion zones (blue) are located on 2-(naphthalen-2-yl)-1*H*-benzo[*d*]imidazole, while in contrast, growth zones (purple) on 2,4-dinitrobenzenosulfonyl part. The presence and change of donor–acceptor substituents does not change the position of these zones. In the case of BIN derivatives, the zones of electron density decay are visible throughout the entire molecule, and the increase zones mainly on naphthalene. Also in this case, the substituent change does not move these zones. On the other hand, the polarity of the environment affects the parameters describing Δ*ρ*(*r*) (Table SI3†). For both BIN-T and BIN derivatives, the amount of the transferred charge drops as a function of solvent polarity. In addition, the value of *q*_CT_ is higher for BIN-T, and the Δ*q*^BIN-T–BIN^_CT_ difference is 0.9 a.u. The same analogy applies to other derivatives. In addition, the introduction of the –NO_2_ substituent increases the value of *q*_CT_ and 
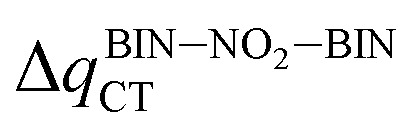
 is 0.5 a.u. The polarity of the environment also significantly affects the charge-transfer distance and decreases it from 1.682 Å in 1,4-Dx to 0.920 Å in water for BIN. In turn, for BIN-T, this quantity increases from 7.503 Å to 8.157 Å. At the same time, BIN-T derivatives have a higher *D*_CT_ value and Δ*D*^BIN-T–BIN^_CT_ = 7.2 Å. The presented analysis indicates the charge-transfer nature of BIN and BIN-T derivatives. It also confirms the contributions from the HOMO–LUMO transition. At the same time, it indicates the possibility of the presence of contributions from other orbitals. Therefore, additional low intensity peaks can be seen in absorption and fluorescence spectra.

The BIN-T molecule is characterized by good solubility in all discussed media. Δ*G*_solv_ increases with increasing solvent polarity, going from −17.62 kcal mol^−1^ in 1.4Dx to −20.37 kcal mol^−1^ in water, with the highest value being reached in MeCN (−29.06 kcal mol^−1^). While similar behavior is observed for other molecules, the introduction of –NHR, –OH, –NO_2_ and –CN significantly improves the solubility of the compound. The presence of the remaining substituents slightly increases the Δ*G*_solv_ value by reducing solubility. Similar conclusions can be drawn after analyzing BIN derivatives. More importantly, the change in structure after thiophenol identification results in a significant reduction in solubility and for BIN Δ*G*^BIN-T–BIN^_solv_ in 1,4Dx is 5.59 kcal mol^−1^ and in water 1.85 kcal mol^−1^. It should also be clearly stated that in terms of Δ*G*_solv_ the desired substituents will be –OH, –NO_2_ and –CN, the presence of which significantly improves the solubility of the compound and thus its optical availability.

### 4.2. Linear optical properties

Theoretical linear optical properties of analyzed markers are shown in Tables 1 and SI5.† Due to the high compatibility of theoretical and experimental values obtained on the basis of PBE0,^18–21^ the spectroscopic parameters were determined using this function. It also accurately predicts BIN-T values experimentally recorded after thiophenol detection. For vertical values, the Δ*λ*^TDDFT-Exp^_ABS_ difference is only 1.63 nm. In the case of BIN-T derivatives, the presence of donor–acceptor substituents results in a shift in absorption maximum (*λ*_ABS_). In the presence of water, donor groups cause bathochromic shift, and acceptor groups cause hypsochromic shift. In the first case, the largest shift of maximum *λ*_ABS_ is a result of the presence of the –NHR group. Considering the effect of substituents on the magnitude of the solvatochromic shift, the analyzed derivatives can be ordered as follows: BIN-T–NHR (31.25 nm) > BIN-T–NH_2_ (24.97 nm) > BIN-T–OR (9.39 nm) > BIN-T–OH (3.94 nm) > BIN-T (0 nm) > BIN-T–CN (−9.60 nm) > BIN-T–NO_2_ (−10.08 nm). Slightly different conclusions can be drawn after analyzing BIN derivatives. In this case, acceptor substituents cause bathochromic shift, while –OH and –OR moves the maximum of *λ*_ABS_ towards shorter wavelengths: BIN–NO_2_ (84.63 nm) > BIN–CN (33.25 nm) > BIN–NH_2_ (21.85 nm) > BIN–NHR (13.58 nm) > BIN (0 nm) > BIN–OH (−6.35 nm) > BIN–OR (−8.62 nm). Moreover, detection of thiophenols and achieving BIN structure induces hypsochromic shift. The highest Δ*λ*^BIN-T–BINABS^_ABS_ values are observed for donor substituents. In –NO_2_ there is a bathochromic shift: –OR (88.86 nm) > –NHR (88.52 nm) > –OH (81.14 nm) > –NH_2_ (73.97 nm) > BIN (70.85 nm) > –CN (28.00 nm) > –NO_2_ (−23.86 nm).

**Table 1:** Linear and nonlinear optical properties in water

	*λ* ^TDDFT^_ABS_	*λ* ^cLR^_ABS_	*λ* ^TDDFT^_EM_	*μ*_GS_	*μ*_CT_	〈*α*〉	Δ*α*	*β*_vec_	〈*δ*^OF^〉	*σ* ^(2)^_OF_
BIN	331.63	330.62	460.31	3.43	7.49	342.96	340.39	1169.43	27.74	0.13
BIN–NH_2_	353.48	350.81	471.48	1.92	4.08	364.64	387.01	2223.18	339.40	1.44
BIN–NHR	345.21	351.50	436.62	5.26	7.05	363.73	307.71	799.39	351.51	1.56
BIN–OH	325.28	334.03	462.75	5.81	6.78	340.17	332.24	1020.66	103.13	0.49
BIN–OR	323.01	322.42	462.65	5.18	7.91	356.04	328.75	514.84	45.17	0.23
BIN–NO_2_	416.26	400.71	540.55	7.87	14.07	359.18	337.84	88.14	217.52	0.91
BIN–CN	364.88	364.84	507.30	8.39	11.18	363.82	350.91	257.54	442.17	1.87
BIN-T	402.48	405.31	582.65	1.83	5.11	443.19	517.69	7.07	3608.61	13.17
BIN-T–NH_2_	427.45	426.44	627.74	2.38	5.22	462.83	540.67	645.13	2872.63	7.47
BIN-T–NHR	433.73	435.31	623.63	3.49	5.97	383.38	539.01	694.93	1806.53	4.95
BIN-T–OH	406.42	409.95	520.00	3.95	5.46	451.19	525.36	2049.36	2197.89	7.14
BIN-T–OR	411.87	416.81	607.05	1.69	4.84	407.28	529.48	1760.67	2599.40	9.76
BIN-T–NO_2_	392.40	390.72	606.48	5.23	10.46	418.86	534.72	2184.23	2403.32	10.27
BIN-T–CN	392.88	394.25	525.50	5.92	10.37	403.73	501.07	1496.24	5864.79	25.33

Considering the effect of the solvent, negative solvatochromism is observed for BIN-T derivatives and the maximum absorption is shifted in the direction of shorter wavelengths with increasing solvent polarity. This effect is observed regardless of the nature of the attached substituent, both for vertical values and the ones determined based on the cLR model. Also, for all BIN derivatives non-monotonic solvatochromism is observed. The excitation energy values *E*_EX_ increase as a function of the polarity of the medium, however the transition from MeCN to DMSO is followed by a slight decrease in *E*_EX_. The only exception from the above results is the BIN–NO_2_ derivative, for which positive solvatochromism is observed.

The interconnectedness of the analysis of spectroscopic parameters with MEP indicates the possibility of specific interactions in the solute–solvent system. A non-monotonic increase in excitation energy indicates on larger polarization and better S_g_ stabilization. However, this is not consistent with the polarity of the excited state (Δ*μ*_CT-g_, eqn (1)). Hypsochromic shift, as the effect of the increase in environmental polarity, should result in the *μ*_g_ > *μ*_CT_ relationship. In contrast, for both BIN-T and BIN derivatives there is an inverse relationship (Table SI6†), which is characteristic of positive solvatochromism. In any case, the polarity of the excited state also changes non-monotonously. In water, for BIN-T derivatives, the highest Δ*μ*_CT-g_ value is characteristic for markers with electron-withdrawing substituents: 
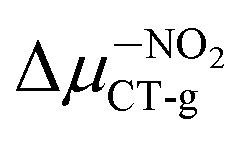
 (5.23 D) > Δ*μ*^–CN^_CT-g_ (4.45 D) > Δ*μ*^BIN-T^_CT-g_ (3.28 D) > Δ*μ*^–OR^_CT-g_ (3.15 D) > 
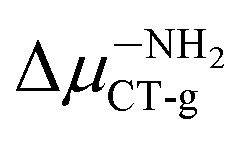
 (2.84 D) > Δ*μ*^–NHR^_CT-g_ (2.48 D) > Δ*μ*^–OH^_CT-g_ (1.51 D). In the case of BIN derivatives, again the presence of –NO_2_ results in the highest polarity of the excited state, however the BIN molecule has a higher Δ*μ*_CT-g_ value than BIN–CN: 
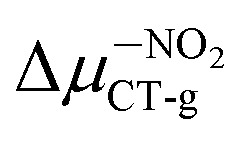
 (6.20 D) > Δ*μ*^BIN^_CT-g_ (4.06 D) > Δ*μ*^–CN^_CT-g_ (2.79 D) > Δ*μ*^–OR^_CT-g_ (2.73 D) > 
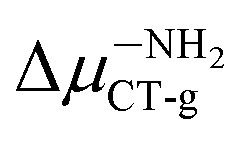
 (2.16 D) > Δ*μ*^–NR^_CT-g_ (1.79 D) > Δ*μ*^–OH^_CT-g_ (0.97 D). In addition, after thiophenol identification, the polarity of the excited state decreases, except for BIN–NO_2_ and BIN.

**Fig. 3:**
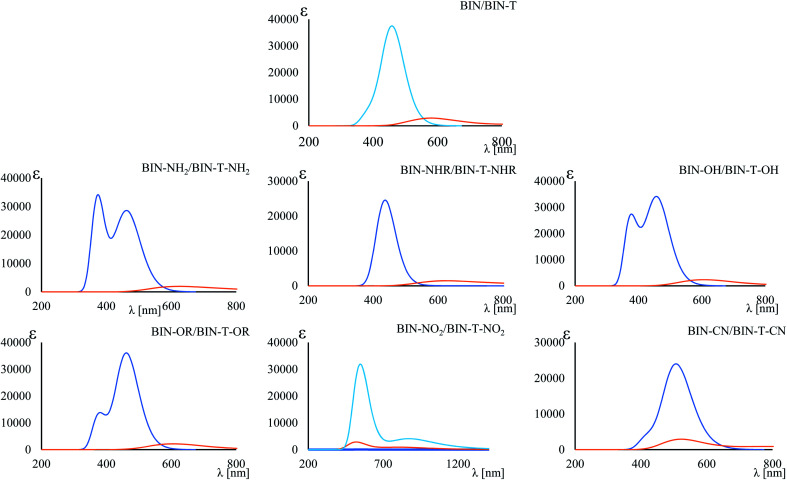
Maximum fluorescence band. Blue line – BIN derivatives, orange line – BIN-T derivatives.

Tables 1 and SI7† show the values of the de-excitation energy (*λ*_FL_). Also in this case the functional PBE0 perfectly reproduces the values and Δ*λ*^TDDFT-Exp^_FL_ is only 0.31 nm. The increasing polarity of the environment causes non-monotonic solvatochromism, both for BIN-T and BIN derivatives. More importantly, thiophenol identification results in a hypsochromic shift at the maximum fluorescence position. The highest value of this shift (Δ*λ*_FL_) is characteristic for donor substituents: Δ*λ*^–NHR^_FL_ (187.01 nm) > 
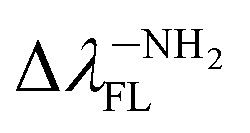
 (156.26 nm) > Δ*λ*^–OR^_FL_ (144.40 nm) > Δ*λ*^BIN^_FL_ (122.34 nm) > 
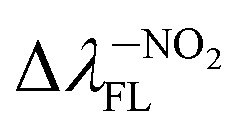
 (65.93 nm) > Δ*λ*^–OH^_FL_ (57.25 nm) > Δ*λ*^–CN^_FL_ (18.20 nm). In addition, in line with experimental relationships, for BIN-T fluorescence intensity is negligible (Fig. 3). Identification of thiophenols and detachment of 2,4-dinitrobenzenesulfonyl group significantly increases the intensity of *λ*_FL_. At the same time, it can be observed that with the exception of BIN and BIN–NHR, a second, less intense fluorescence band appears shifted towards shorter wavelengths. In general, the used substituents significantly shift the maximum *λ*_FL_ towards longer wavelengths, and only the –NHR substituent results in hypochromic shift relative to BIN: 
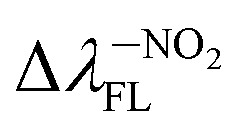
 (80.24 nm) > Δ*λ*^–CN^_FL_ (46.99 nm) > 
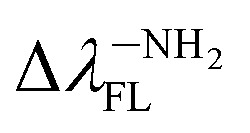
 11.17 nm) > Δ*λ*^–OH^_FL_ (2.44 nm) > Δ*λ*^–OR^_FL_ (2.34 nm) > Δ*λ*^BIN^_FL_ (0 nm) > Δ*λ*^–NHR^_FL_ (−23.59 nm).

All BIN derivatives have a high Stokes shift value (Δ*ν*^St^). In each case, its value increases monotonously as a function of the solvent polarity. However, while for *λ*_ABS_ and *λ*_FL_ donor and acceptor substituents have some relationship, in this case there is none. For water, the largest Δ*ν*^St^ values are found for BIN–OH and BIN–OR derivatives, which are 9132.80 cm^−1^ and 9344.82 cm^−1^, respectively. The lowest value is observed for BIN–NO_2_, amounting to 5523.77 cm^−1^. By ordering these derivatives in terms of Δ*ν*^St^*versus* BIN: BIN–OR (914.58 cm^−1^) > BIN–OH (703.21 cm^−1^) > BIN (0 cm^−1^) > BIN–CN (−735.53 cm^−1^) > BIN–NH_2_ (−1349.26 cm^−1^) > BIN–NHR (−2364.93 cm^−1^) > BIN–NO_2_ (−2905.83 cm^−1^), it can be concluded that the presence of the –OH and –OR substituents maximizes the linear response, thus increasing their detection capabilities. Although all derivatives are described by a high Δ*ν*^St^ value, the presence of –NO_2_ will be the least desirable substituent in the marker structure.

### 4.3. Nonlinear opical properties

The polarizability (Δ*α*, eqn (3)) and first hyperpolarizability (*β*_vec_, eqn (4)) of molecules irradiated with intense laser light giving the electric field is the subject of many research in terms of understanding various nonlinear optical properties (NLO). In particular, these studies include the interrelationship of NLO with the electronic structure to design new multifunctional fluorescence probes. The calculated values of Δ*α* and *β*_vec_ are collected in Tables 1 and SI8.† Firstly, for both BIN and BIN-T derivatives, the Δ*α* and *β*_vec_ values depend on the presence of the solvent. They increase monotonously as a function of the polarity of the medium. In addition, the polarizability values for BIN-T derivatives are on average 150 a.u. higher than BIN. In the case of the first hyperpolarizability, it depends on the structure of the probe and *e.g. β*^BIN^_vec_ > *β*^BIN-T^_vec_, 
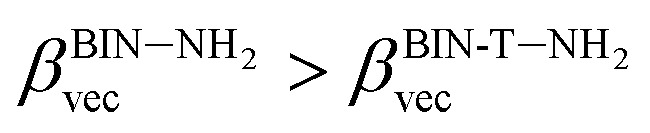
, *β*^BIN-T–NHR^_vec_ > *β*^BIN–NHR^_vec_, *β*^BIN-T–OR^_vec_ > *β*^BIN–OR^_vec_*etc.* More importantly, changing substituents causes changes in the nonlinear response of the system. In the case of Δ*α*, if the values are similar, the analyzed compounds can be ordered in the following way: BIN-T–NH_2_ > BIN-T–NHR > BIN-T–NO_2_ > BIN-T–OR > BIN-T–OH > BIN-T > BIN-T–CN and BIN–NH_2_ > BIN–CN > BIN–NHR > BIN–NO_2_ > BIN–OR > BIN > BIN–OH. In the case of *β*_vec_, these series are as follows: BIN-T–NO_2_ > BIN-T–OH > BIN-T–OR > BIN-T–CN > BIN-T–NHR > BIN-T–NH_2_ > BIN-T and BIN–NH_2_ > BIN > BIN–OH > BIN–NHR > BIN–OR > BIN–CN > BIN–NO_2_. Since the non-linear response will be derived from the structure obtained after thiophenol identification, the factor enhancing the NLO response will be the presence of the –NH_2_ group and can be efficient in Second Harmonic Generation (SHG).

Tables S1 and SI9† show the value of two-photon absorption cross section (TPA, 〈*δ*^OF^〉 eqn (6)). In general, it is difficult to decisively conclude about the effect of the solvent on the 〈*δ*^OF^〉 value. Non-monotonic solvatochromism is observed for BIN-T, whereas negative solvatochromism is observed for BIN. Positive solvatochromism is revealed in the values describing BIN–NH_2_ and BIN-T–OR. In addition, the values for BIN-T derivatives are on average 3.000 a.u. higher than for BIN. However, in order to compare the calculated values of the TPA with those determined experimentally (*σ*^(2)^_OF_, eqn (5)), the relation (5) was used. In this equation, the broadening of the final state due to its finite lifetime 0.25 eV was assumed. The effect of the solvent on the *σ*^(2)^_OF_ value remains the same as for 〈*δ*^OF^〉. More importantly, although BIN-T derivatives have a higher *σ*^(2)^_OF_ value, none of the analyzed markers meet the requirements for use in two-photon imaging. BIN-T–CN (25.33 GM) has the highest value, while BIN (0.13 GM) has the lowest one. Thus, these values indicate a very poor NLO response signal. In the case of BIN-T derivatives, only the presence of the –CN substituent is a factor increasing the non-linear response. The remaining substituents reduce the *σ*^(2)^_OF_ value, with the highest minimization observed for –NHR. For BIN derivatives, each substituent increases the two-photon absorption cross section value. The largest increase is observed for BIN–NH_2_, –NHR and –CN. Based on this analysis, to obtain a probe with a high TPA value, used as tools in real-time dynamic *in vivo* and *in vitro* research, an additional chromophore group intensifying the NLO response should be added to the structure of the analyzed dyes.

### 4.4. AutoDock simulations

**Fig. 4:**
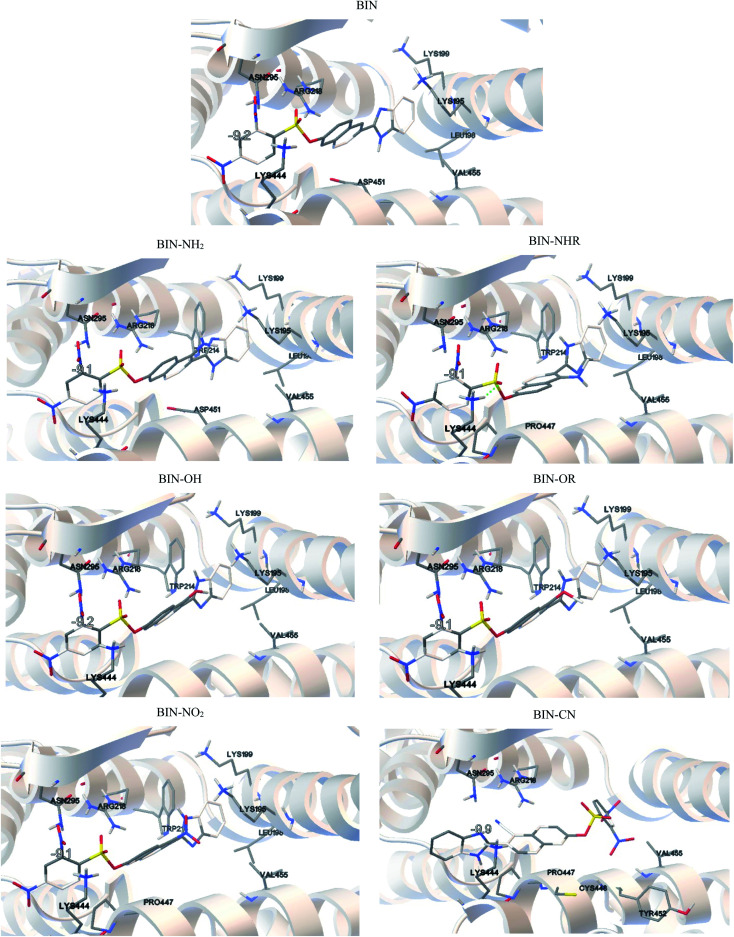
Results of the AutoDock simulations.

BIN-T is a probe for detecting thiophenols in solutions. As mentioned earlier (Scheme 1), the identification reaction occurs by 6-(1*H*-benzimidazole) −2 (2,4-dinitrobenzenesulfonate) naphthalene reacting with thiophenol sulfur. Therefore, this marker can also be an alternative as a fluorescent probe used in medical imaging. In this case, protein conjugation occurs by reaction with cysteine. Human Serum Albuminum (HSA) was selected for this study. For BIN-T derivatives, the presence of a substituent or its change does not alter the site of active binding to the protein. In each case, conjugation to HSA occurs *via* CYS448 (Fig. 4). The BIN-T–CN marker has the highest affinity for this active center, for which binding energy (Δ*G*_b_) is −9.9 kcal mol^−1^ (Table SI10†). This active binding site has an inhibition constant (*K*_i_) of 1.00 μM. The BIN-T–CN is inserted into the aromatic cage formed by CYS448, ASN295, ARG218, LYS444, PRO447, TYR452 and VAL455. In this active site, no π–π* interactions occur and the system is not stabilized by the presence of a H-bond. BIN-T–OH and BIN-T have slightly lower affinity for HSA and Δ*G*_b_ = −9.2 kcal mol^−1^. Inhibition constant is 1.57 μM and 2.53 μM, respectively. The active impact cavity here is: LYS199, LYS195, LEU198, VAL455, ASN295, ARG218 and LYS444. In the case of BIN-T–OH, TRP214 also interacts here and in BIN-T – ASP451. Also in this case no hydrogen bonds are observed and no π–π* interactions. For other probes Δ*G*_b_ is −9.1 kcal mol^−1^. Only for BIN-T–NHR a hydrogen bond is formed between the oxygen of the sulfone group and the hydrogen of LYS444. In addition, the lowest *K*_i_ value of 0.63 μM was recorded for BIN-T–NO_2_.

During conjugation, the O–S bond raptures and then a new one forms with the protein. The speed of biocomplex creation will therefore also be affected by the energy barrier (Δ*E*) necessary to overcome. According to Fig. SI1,† the lowest Δ*E* value when moving the oxygen atom away from the sulfur atom at a distance of 2.5 Å is observed for BIN-T–OR and is 14.5 kcal mol^−1^. A slightly higher value is observed for BIN-T-OH, for which Δ*E* = 16.87 kcal mol^−1^. The strongest bond, and thus hindering the conjugation, is the O–S in BIN–CN, where Δ*E* = 26.63 kcal mol^−1^.

### 4.5. Biological activities

The BIN-T derivatives are described by relatively good bioavailability (log *P* > 5). Only for BIN-T–NHR this value drops slightly and amounts to 4.70 ± 0.25. This suggests good permeability through cell membranes and achieving adequate concentration at the site of interaction with thiophenols. In turn, BIN derivatives are described with a log *P* value in the range of 3–4. The exceptions are BIN–NH_2_ and BIN–NHR, for which log *P* < 3. The log BCF value calculated for BIN-T in the range from −4.2 to −4.5 indicates the lack of bioaccumulation in the tissues of living organisms and the ease of excretion with urine. After identifying thiophenols, log BCF decreases by one unit (in the range from −3.0 to −3.3). Despite this, BIN derivatives should not bioaccumulate after fulfilling their optical role. In addition, both BIN-T and BIN derivatives are characterized by high metabolism by CYP450-2D6 and CYP450-3A4 (probability above 80%) (Tables SI11 and SI12†). This indicates that both forms of markers will be rapidly excreted from tissues without interacting with other biomolecules and drugs.

**Fig. 5:**
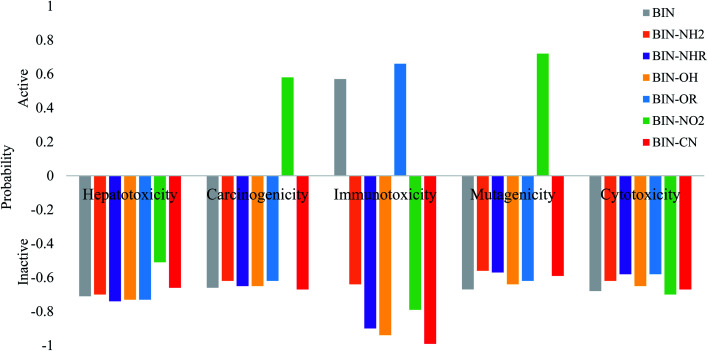
Theoretically determined toxicological parameters.

The calculated oral toxicity value is LD_50_ > 1500 mg kg^−1^. Therefore BIN-T and BIN derivatives should be classified in class 4 in terms of the degree of toxicity and can be considered as practically nontoxic for humans. While the presence and change of a substituent does not affect the toxicity of presented molecules, it does, however, affect other toxicological parameters (Fig. 5). None of the derivatives shows hepatotoxicity and cytotoxicity. The presence of the NO_2_ substituent causes carcinogenicity (probability *P* = 58%) and mutagenicity (*P* = 72%). The –OR group may affect the occurrence of immunotoxicity (*P* = 66%). Immunotoxicity can elicit the reference BIN marker (*P* = 57%). Other probes should not cause any toxic effects. Thus, the introduction of a substituent into the BIN structure eliminates the possible toxic effects of the molecule. In addition, both BIN-T and BIN derivatives have other biological activities suggesting their potential use in other areas of medicine (Tables SI11 and SI12†). First of all, BIN-T derivatives are characterized by high antioxidant activity (*P*> 48%), in particular BIN-T–CN (*P* = 90%). For BIN derivatives probability of this activity drops to zero, except for BIN–NHR (*P* = 27%) and BIN–OR (*P* = 39%). In turn, for BIN-T derivatives no antibacterial activity is observed, while it can be attributed to BIN derivatives, in particular BIN–OH (*P* = 79%), BIN–NO_2_ (*P* = 79%) and BIN–CN (*P* = 80%). The following tables indicate the occurrence of many other activities, such as: alpha-radioprotector, analgetic, anti-psychotic activity diazepine site, anti-tumor alkylic, anti-tumor antimiotic, HIV1-proteaze inhibitory activities and many others. At the same time, the influence of subsequent substituents on the maximization or reduction of these activities is demonstrated there.

## 5 Conclusion

The manuscript presents previously unavailable observations on the influence of donor–acceptor substituents (D/A) on optical parameters and biological activity of the fluorescent probe used to identify thiophenols. This type of research is the first for the derivatives in question and is valuable information for researchers synthesizing new compounds on the benzimidazole backbone. It has been demonstrated that the used D/A substituents do not significantly affect the position of HOMO–LUMO orbitals and only slightly affect the EGAP value. They also do not change the sites for nucleophilic and electrophilic attack and density depletion/increment zones upon photoexcitation. However, it has been shown that the –NO_2_ substituent increases the amount of transferred charge and the charge-transfer distance. Studies have shown that the introduction of the –OH, –NO_2_ and –CN groups into the marker structure significantly improves the solubility and thus the optical availability of the probe. The used substituents cause the solvatochromic shift of the absorption and fluorescence maxima with respect to the experimentally tested marker, and the solvatochromic relationships depend on the structure before and after identification of thiophenols. These observations is extremely important for scientists synthesizing and studying benzimidazole derivatives. Taking into account the Stokes shift value, it can be concluded that the presence of the –OH and –OR substituents maximizes the linear response and increasing detection of the marker. However, the reducing factor will be the presence of –NO_2_. On the other hand, the –NO_2_ substituent maximizes the non-linear response. Nevertheless, the presence of –OR and –OH also enhances NLO values relative to pure dye. The tested derivatives are not used in two-photon imaging. However, this observation may be a clue to search for other substituents enhancing the two-photon story. The analyzed structures show high affinity for HSA, and a change in the D/A substituent slightly reduces binding energy. Therefore, the presented compounds can be used with great success in *in vivo* and *in vitro* tests as fluorescent probes for detecting specific biomolecules containing the –SH group. Therefore, the considered derivatives constitute valuable compounds for the pharmaceutical and medical market. At the same time, the –OR group minimizes the energy barrier necessary to break the bond between the chromophore and the linker, which speeds up and facilitates accessibility during conjugation with protein. All derivatives are characterized by good permeability through cell membranes and no bioaccumulation. A very valuable observation is the fact that, toxicity studies excluded the attractiveness of using the –NO_2_ substituent in *in vivo* and *in vitro* studies due to the potential for carcinogenicity and mutagenicity. By modulating the appropriate D/A substituents on the BIN structure, many valuable drugs can be obtained in the treatment of cancer, HIV, oxidative stress regulation, *etc.* Therefore, the conducted research indicates the high value of these derivatives in medicine. Considering all the above analyzes, it should be clearly stated that the presence of the –OR and –OH substituents significantly maximizes the linear and non-linear optical response of the molecule used as a fluorescent probe in medical imaging.

## Abbreviations and notations

1,4-Dx: 1,4-Dioxane
MeCN: Acetonitrile
DMSO: Dimethylsulfoxide
CH_2_Cl_2_: Dichloromethane
EtOAc: Ethyl acetate
PetEth: Petroleum ether
CT: Charge-transfer excitation
Δ*G*_solv_: Free energies of solvation
G: Ground state
HSA: Human serum albuminum
Δ*G*_b_: Binding energy
*K*_i_
: Inhibition constant
Δ*E*: Energy barrier
log *P*: Octanol–water partition coefficients
CYP2D6: Cytochrome P450-2D6
CYP450-3A4: Cytochrome CYP450-3A4
*P*
: Probability of occurrence
LD_50_: Lethal dose, determination of the toxicity of the substance

## Conflicts of interest

The authors declare that there are no potential financial or non-financial conflicts of interest.

## Supplementary Material

RA-010-D0RA04835F-s001
