# Supplementary material for: Optimizing the optical and biological properties of 6-(1H-benzimidazole)-2-naphthalenol as a fluorescent probe for the detection of thiophenols: a theoretical study
Source: RSC Adv. 2020 Jun 25;10(41):24374–85. doi: 10.1039/d0ra04835f (PMC9122578; doi:10.1039/d0ra04835f)
Supplement: RA-010-D0RA04835F-s001 [file RA-010-D0RA04835F-s001.pdf]

Electronic Supplementary Material (ESI) for RSC Advances.
This journal is © The Royal Society of Chemistry 2020

Optimizing the optical and biological properties of 6-(1H-benzimidazole)-2-naphthalenol as fluorescent probe for the detection of thiophenols: a theoretical study

Przemysław Krawczyk[1*]

[1]Nicolaus Copernicus University, Collegium Medicum, Faculty of Pharmacy, Department of Physical Chemistry, Kurpińskiego 5, 85-950 Bydgoszcz, Poland

*Corresponding author: przemekk@cm.umk.pl; tel: +48 52 5853679

**Table of Contents**	**Page**
**Figure 1.** The result of the O-S bond scan	2
**Table SI1.** The frontier orbital energies in selected solvents for BIN-T derivatives. All values are given in eV	3
**Table SI2.** The frontier orbital energies in selected solvents for BIN derivatives. All values are given in eV	5
**Table SI3.** CT parameters for the bright low-lying excited state	6
**Table SI4.** Free energies (ΔG_{solv}, kcal/mol) of solvation	7
**Table SI5.** The vertical and cLR corrected excitation energies (in nm)	7
**Table SI6.** Calculated values of dipole moments (in D) for the ground and CT excited state	8
**Table SI7.** The vertical de-excitation energies (in nm)	8
**Table SI8.** Nonlinear properties. Values are given in a.u.	9
**Table SI9.** Results of the two-photon absorption cross section calculation	9
**Table SI10**. Binding free energies (ΔG_b, kcal/mol) obtained during AutoDock simulations	10
**Table SI11.** Biological activities for BIN-T derivatives	11
**Table SI12.** Biological activities for BIN derivatives	12

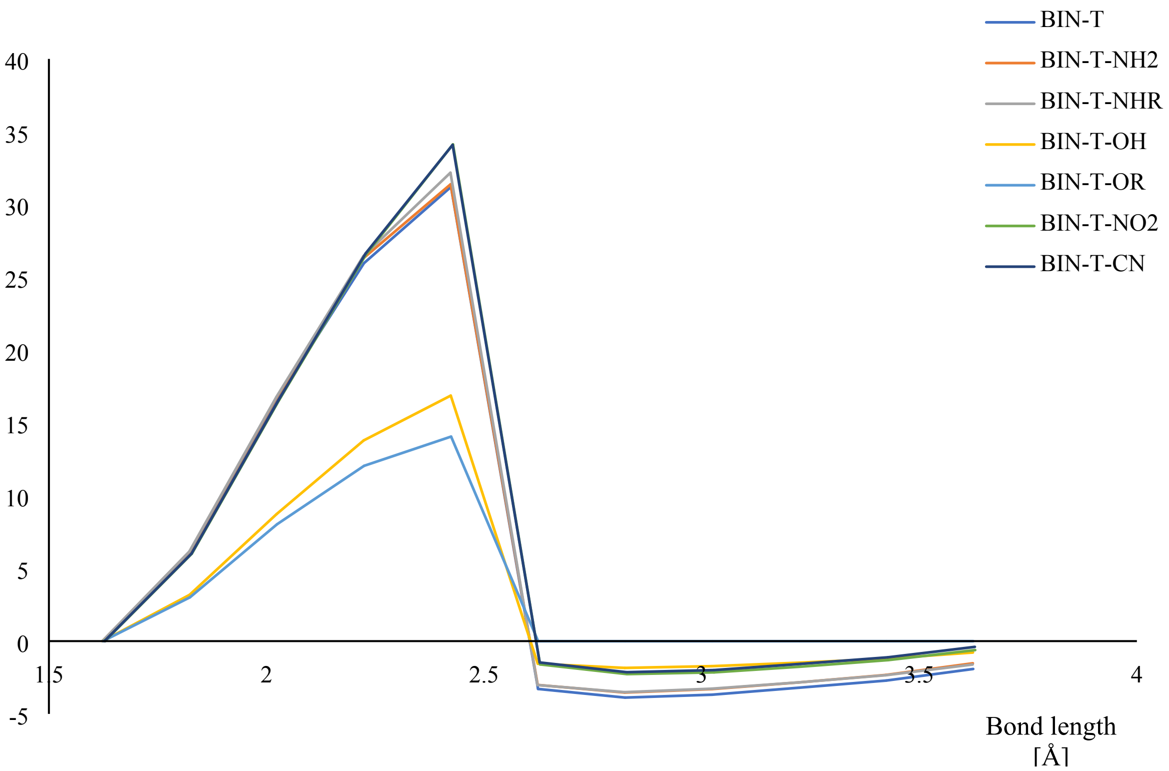

Figure 1. The result of the O-S bond scan

Table SI1. The frontier orbital energies in selected solvents for BIN-T derivatives. All values are given in eV

BIN-T	1.4-Dx	MeCN	DMSO	Water
E_{HOMO}	-6.2820	-6.3536	-6.3615	-6.3645
E_{LUMO}	-3.6755	-3.5664	-3.5639	-3.5612
E_{GAP}	2.6065	2.7872	2.7976	2.8033
η	1.3033	1.3936	1.3988	1.4016
μ	-4.9788	-4.9600	-4.9627	-4.9629
χ	4.9788	4.9600	4.9627	4.9629
BIN-T-NH_2				
E_{HOMO}	-5.7081	-5.7740	-5.7761	-5.7780
E_{LUMO}	-3.6554	-3.5620	-3.5598	-3.5579
E_{GAP}	2.0527	2.2119	2.2163	2.2201
η	1.0264	1.1060	1.1081	1.1101
μ	-4.6817	-4.6680	-4.6680	-4.6680
χ	4.6817	4.6680	4.6680	4.6680
BIN-T-NHR				
E_{HOMO}	-5.8050	-5.8055	-5.8083	-5.8118
E_{LUMO}	-3.6388	-3.5571	-3.5555	-3.5530
E_{GAP}	2.1662	2.2484	2.2528	2.2587
η	1.0831	1.1242	1.1264	1.1294
μ	-4.7219	-4.6813	-4.6819	-4.6824
χ	4.7219	4.6813	4.6819	4.6824
BIN-T-OH				
E_{HOMO}	-6.0804	-6.1449	-6.1468	-6.1498
E_{LUMO}	-3.6516	-3.5656	-3.5637	-3.5609
E_{GAP}	2.4288	2.5793	2.5831	2.5889
η	1.2144	1.2897	1.2916	1.2944
μ	-4.8660	-4.8552	-4.8552	-4.8554
χ	4.8660	4.8552	4.8552	4.8554
BIN-T-OR				
E_{HOMO}	-6.3612	-6.4214	-6.4236	-6.4268
E_{LUMO}	-3.6788	-3.5645	-3.5620	-3.5590
E_{GAP}	2.6825	2.8569	2.8615	2.8678
η	1.3412	1.4285	1.4308	1.4339
μ	-5.0200	-4.9929	-4.9928	-4.9929
χ	5.0200	4.9929	4.9928	4.9929
BIN-T-NO_2				
E_{HOMO}	-6.6511	-6.7098	-6.7118	-6.7145
E_{LUMO}	-3.7512	-3.5881	-3.5849	-3.5805

E_{GAP}	2.8999	3.1217	3.1269	3.1339
η	1.4500	1.5609	1.5634	1.5670
μ	-5.2011	-5.1490	-5.1483	-5.1475
χ	5.2011	5.1490	5.1483	5.1475
BIN-T-CN				
E_{HOMO}	-6.6179	-6.6832	-6.6854	-6.6883
E_{LUMO}	-3.7433	-3.5846	-3.5816	-3.5767
E_{GAP}	2.8746	3.0986	3.1037	3.1116
η	1.4373	1.5493	1.5519	1.5558
μ	-5.1806	-5.1339	-5.1335	-5.1325
χ	5.1806	5.1339	5.1335	5.1325

Table SI2. The frontier orbital energies in selected solvents for BIN derivatives. All values are given in eV

BIN	1.4-Dx	MeCN	DMSO	Water
E_{HOMO}	-5.9506	-6.0583	-6.0608	-6.0646
E_{LUMO}	-1.6062	-1.6990	-1.7014	-1.7044
E_{GAP}	4.3444	4.3594	4.3594	4.3602
η	2.1722	2.1797	2.1797	2.1801
μ	-3.7784	-3.8787	-3.8811	-3.8845
χ	3.7784	3.8787	3.8811	3.8845
BIN-NH_2				
E_{HOMO}	-5.4882	-5.6074	-5.6104	-5.6139
E_{LUMO}	-1.3395	-1.4448	-1.4472	-1.4508
E_{GAP}	4.1488	4.1626	4.1632	4.1632
η	2.0744	2.0813	2.0816	2.0816
μ	-3.4138	-3.5261	-3.5288	-3.5324
χ	3.4138	3.5261	3.5288	3.5324
BIN-NHR				
E_{HOMO}	-5.5258	-5.6545	-5.6580	-5.6545
E_{LUMO}	-1.2638	-1.3512	-1.3536	-1.3512
E_{GAP}	4.2620	4.3033	4.3044	4.3033
η	2.1310	2.1517	2.1522	2.1517
μ	-3.3948	-3.5028	-3.5058	-3.5028
χ	3.3948	3.5028	3.5058	3.5028
BIN-OH				
E_{HOMO}	-5.9027	-6.0118	-6.0145	-6.0183
E_{LUMO}	-1.5006	-1.5485	-1.5504	-1.5528
E_{GAP}	4.4021	4.4633	4.4642	4.4655
η	2.2011	2.2317	2.2321	2.2328
μ	-3.7016	-3.7801	-3.7825	-3.7856
χ	3.7016	3.7801	3.7825	3.7856
BIN-OR				
E_{HOMO}	-5.9753	-6.1052	-6.1087	-6.1136
E_{LUMO}	-1.5101	-1.6119	-1.6138	-1.6162
E_{GAP}	4.4652	4.4933	4.4949	4.4974
η	2.2326	2.2466	2.2475	2.2487
μ	-3.7427	-3.8585	-3.8612	-3.8649
χ	3.7427	3.8585	3.8612	3.8649
BIN-NO_2				
E_{HOMO}	-6.3721	-6.4383	-6.4404	-6.4431
E_{LUMO}	-2.3194	-2.5543	-2.5611	-2.5706

E_{GAP}	4.0527	3.8840	3.8793	3.8725
η	2.0263	1.9420	1.9397	1.9363
μ	-4.3458	-4.4963	-4.5008	-4.5069
χ	4.3458	4.4963	4.5008	4.5069
BIN-CN				
E_{HOMO}	-6.3457	-6.4140	-6.4159	-6.4184
E_{LUMO}	-2.3069	-2.3752	-2.3769	-2.3793
E_{GAP}	4.0388	4.0388	4.0391	4.0391
η	2.0194	2.0194	2.0195	2.0195
μ	-4.3263	-4.3946	-4.3964	-4.3988
χ	4.3263	4.3946	4.3964	4.3988

Table SI3. CT parameters for the bright low-lying excited state

	1,4-Dx		MeCN		DMSO		Water	
	q_{CT}	D_{CT}	q_{CT}	D_{CT}	q_{CT}	D_{CT}	q_{CT}	D_{CT}
BIN	0.426	1.682	0.404	0.956	0.403	0.880	0.403	0.920
BIN-NH_2	0.462	0.413	0.470	0.660	0.471	0.708	0.470	0.685
BIN-NHR	0.578	2.136	0.556	1.968	0.554	1.945	0.555	1.950
BIN-OH	0.523	2.930	0.445	2.334	0.441	2.244	0.441	2.271
BIN-OR	0.384	1.088	0.382	0.770	0.382	0.716	0.382	0.734
BIN-NO_2	0.891	1.331	0.960	1.534	0.960	1.541	0.962	1.550
BIN-CN	0.559	2.008	0.544	1.208	0.544	1.187	0.544	1.150
BIN-T	1.351	7.503	1.305	8.164	1.305	8.151	1.304	8.157
BIN-T -NH_2	1.371	6.857	1.318	7.550	1.317	7.558	1.316	7.578
BIN-T -NHR	1.399	6.576	1.347	7.182	1.346	7.196	1.345	7.212
BIN-T -OH	1.359	6.829	1.310	7.625	1.309	7.582	1.308	7.598
BIN-T -OR	1.364	7.178	1.316	7.764	1.315	7.761	1.313	7.792
BIN-T -NO_2	1.377	7.909	1.315	8.637	1.314	8.644	1.313	8.656
BIN-T -CN	1.367	7.861	1.310	8.608	1.309	8.616	1.308	8.631

Table SI4. Free energies (ΔG_{solv}, kcal/mol) of solvation

	1,4-Dx	MeCN	DMSO	Water
BIN	-12.03	-23.72	-20.21	-18.52
BIN-NH_2	-12.01	-24.52	-21.10	-18.92
BIN-NHR	-13.42	-27.28	-23.52	-22.15
BIN-OH	-15.14	-28.97	-25.46	-25.70
BIN-OR	-11.66	-25.47	-21.67	-20.64
BIN-NO_2	-14.68	-27.95	-24.51	-21.54
BIN-CN	-13.65	-28.23	-24.50	-22.48
BIN-T	-17.62	-29.06	-26.48	-20.37
BIN-T-NH_2	-17.70	-30.59	-28.75	-23.30
BIN-T-NHR	-19.74	-34.82	-31.70	-27.76
BIN-T-OH	-19.81	-31.05	-28.15	-20.88
BIN-T-OR	-16.84	-29.94	-26.53	-20.28
BIN-T-NO_2	-20.05	-34.63	-30.28	-23.65
BIN-T-CN	-20.17	-35.09	-31.70	-24.78

Table SI5. The vertical and cLR corrected excitation energies (in nm)

	1.4-Dx			MeCN			DMSO			Water		
	λ_{ABS}^{TDDFT}	f	λ_{ABS}^{cLR}	λ_{ABS}^{TDDFT}	f	λ_{ABS}^{cLR}	λ_{ABS}^{TDDFT}	f	λ_{ABS}^{cLR}	λ_{ABS}^{TDDFT}	f	λ_{ABS}^{cLR}
BIN	334.11	0.5319	330.62	331.81	0.5849	327.97	332.60	0.6165	328.04	331.63	0.5831	327.86
BIN-NH_2	355.99	0.4503	350.81	353.67	0.5063	349.06	354.62	0.5316	349.22	353.48	0.5054	349.00
BIN-NHR	350.60	0.0793	351.50	345.56	0.0888	346.19	345.93	0.0934	346.51	345.21	0.0889	345.81
BIN-OH	332.50	0.1317	334.03	325.59	0.2252	324.65	325.99	0.2456	324.64	325.28	0.2304	324.25
BIN-OR	325.69	0.3753	322.42	323.16	0.3648	320.33	323.59	0.3831	320.28	323.01	0.3645	320.23
BIN-NO_2	386.47	0.0972	400.71	414.60	0.0485	438.82	415.52	0.0503	443.64	416.26	0.0470	440.58
BIN-CN	365.28	0.3927	364.84	364.97	0.3802	363.98	365.73	0.3941	364.50	364.88	0.3770	363.90
BIN-T	428.75	1.0002	422.29	403.90	1.0001	407.43	403.17	1.0001	408.65	402.48	1.0001	405.31
BIN-T-NH_2	453.15	1.0015	406.48	428.62	1.0018	428.09	428.00	1.0019	426.97	427.45	1.0018	426.44
BIN-T-NHR	472.93	1.0009	472.15	435.34	1.0011	476.89	434.67	1.0011	435.99	433.73	1.0011	435.31
BIN-T-OH	424.19	1.0011	429.29	407.45	1.0013	407.92	407.03	1.0001	409.85	406.42	1.0013	409.95
BIN-T-OR	440.23	1.0001	445.34	413.14	1.0000	414.86	412.55	1.0002	417.54	411.87	1.0000	416.81
BIN-T-NO_2	424.52	1.0003	422.64	393.58	1.0001	429.33	392.87	1.0001	397.43	392.40	1.0698	390.72
BIN-T-CN	422.93	1.0003	426.18	394.37	1.0003	425.82	393.77	1.0003	393.99	392.88	1.0003	394.25

Table SI6. Calculated values of dipole moments (in D) for the ground and CT excited state

	1,4-Dx		MeCN		DMSO		Water	
	μ_{GS}	μ_{CT}	μ_{GS}	μ_{CT}	μ_{GS}	μ_{CT}	μ_{GS}	μ_{CT}
BIN	2.67	8.67	3.39	7.47	3.41	7.06	3.43	7.49
BIN-NH_2	1.58	2.89	1.90	4.04	1.91	4.21	1.92	4.08
BIN-NHR	4.09	6.04	5.18	6.98	5.22	7.00	5.26	7.05
BIN-OH	4.84	8.02	5.76	6.75	5.79	6.62	5.81	6.78
BIN-OR	3.67	5.91	5.11	7.83	5.16	7.56	5.18	7.91
BIN-NO_2	6.19	10.03	7.78	13.87	7.82	13.94	7.87	14.07
BIN-CN	6.55	11.75	8.32	11.17	8.34	11.15	8.39	11.18
BIN-T	1.32	4.90	2.16	5.13	1.81	5.11	1.83	5.11
BIN-NH_2	2.51	4.64	2.77	4.84	2.79	5.17	2.38	5.22
BIN-NHR	3.06	4.71	3.50	4.84	3.49	5.96	3.49	5.97
BIN-OH	3.47	10.88	3.90	5.04	3.92	5.05	3.95	5.46
BIN-OR	1.12	4.71	1.62	4.79	1.65	4.82	1.69	4.84
BIN-NO_2	3.88	5.41	5.24	9.85	5.27	10.21	5.23	10.46
BIN-CN	4.44	5.76	5.84	10.18	5.88	10.31	5.92	10.37

Table SI7. The vertical de-excitation energies (in nm)

	1,4-Dx	MeCN	DMSO	Water
BIN	459.88	460.64	462.41	460.31
BIN-NH_2	439.93	471.84	473.24	471.48
BIN-NHR	439.59	436.88	437.86	436.62
BIN-OH	471.62	462.85	464.71	462.75
BIN-OR	464.7	462.94	464.64	462.65
BIN-NO_2	516.55	538.98	539.65	540.55
BIN-CN	501.59	507.14	509.03	507.30
BIN-T	693.23	583.30	584.69	582.65
BIN-T-NH_2	715.55	625.50	626.52	627.74
BIN-T -NHR	730.02	629.82	627.44	623.63
BIN-T -OH	665.85	863.21	857.92	520.00
BIN-T -OR	730.62	612.24	610.00	607.05
BIN-T -NO_2	696.19	609.17	607.50	606.48
BIN-T -CN	668.50	516.33	524.55	525.50

Table SI8. Nonlinear properties. Values are given in a.u.

	1,4-Dx		MeCN		DMSO		Water	
	$\Delta\alpha$	β_{vec}	$\Delta\alpha$	β_{vec}	$\Delta\alpha$	β_{vec}	$\Delta\alpha$	β_{vec}
BIN	284.82	581.46	340.03	1140.92	341.31	1153.5	342.96	1169.43
BIN-NH_2	301.13	1365.42	361.43	2182.52	362.82	2200.17	364.64	2223.18
BIN-NHR	301.96	492.60	360.57	786.91	361.93	792.37	363.73	899.39
BIN-OH	282.72	266.42	337.23	979.07	338.50	997.04	340.17	1020.66
BIN-OR	297.61	253.73	353.17	501.68	354.41	507.41	356.04	514.84
BIN-NO_2	296.82	255.92	356.02	58.87	357.38	71.34	359.18	88.14
BIN-CN	300.59	140.96	360.62	226.14	362.00	239.55	363.82	257.54
BIN-T	515.28	42.44	515.28	21.65	513.95	47.09	517.69	7.07
BIN-T-NH_2	455.37	1252.42	536.07	673.81	538.02	667.00	540.67	645.13
BIN-T-NHR	454.13	807.11	534.42	686.67	536.42	690.26	539.01	694.93
BIN-T-OH	444.10	1598.86	521.08	2034.87	522.92	2041.02	525.36	2049.36
BIN-T-OR	445.89	1686.79	525.05	1703.11	526.99	1726.79	529.48	1760.67
BIN-T-NO_2	449.52	1754.66	529.87	2121.35	531.81	2121.67	534.72	2184.23
BIN-T-CN	410.21	1423.30	498.73	1461.47	500.53	1479.39	501.07	1496.24

Table SI9. Results of the two-photon absorption cross section calculation

	1,4-Dx		MeCN		DMSO		Water	
	$\langle\delta^{OF}\rangle$	σ_{OF}^2	$\langle\delta^{OF}\rangle$	σ_{OF}^2	$\langle\delta^{OF}\rangle$	σ_{OF}^2	$\langle\delta^{OF}\rangle$	σ_{OF}^2
BIN	29.07	0.14	28.27	0.14	27.83	0.13	27.74	0.13
BIN-NH_2	327.70	1.39	337.78	1.43	339.40	1.44	339.40	1.44
BIN-NHR	357.12	1.57	351.51	1.56	351.51	1.56	351.51	1.56
BIN-OH	27.78	0.03	103.13	0.49	103.13	0.49	103.31	0.49
BIN-OR	68.98	0.35	46.03	0.23	45.73	0.23	45.17	0.23
BIN-NO_2	219.14	0.92	215.55	0.90	216.27	0.90	217.52	0.91
BIN-CN	424.68	1.81	442.17	1.87	442.17	1.87	442.17	1.877
BIN-T	3130.15	11.68	3629.59	13.25	3607.64	13.17	3608.61	13.17
BIN-T-NH_2	2606.99	7.01	2766.81	7.24	2763.50	7.24	2872.63	7.47
BIN-T-NHR	1780.08	4.88	1783.85	4.89	1791.41	4.91	1806.53	4.95
BIN-T-OH	1988.34	6.61	2202.27	7.15	2200.92	7.15	2197.89	7.14
BIN-T-OR	2035.53	7.90	2545.60	9.55	2568.09	9.64	2599.40	9.76
BIN-T-NO_2	1914.70	8.31	2398.60	10.25	2397.50	10.25	2403.32	10.27
BIN-T-CN	5524.42	24.11	5836.48	25.20	5826.43	25.16	5864.79	25.33

Table SI10. Binding free energies (ΔG_b, kcal/mol) obtained during AutoDock simulations

CYS	BIN-T	BIN-T-NH2	BIN-T-NHR	BIN-T-OH	BIN-T-OR	BIN-T-NO2	BIN-T-CN
34	-4.9	-5.4	-3.6	-5.5	-3.9	-3.9	-5.5
53	-5.6	-5.5	-4.2	-5.5	-4.8	-4.8	-5.3
62	-5.3	-4.7	-4.8	-5.6	-4.1	-4.1	-4.6
75	-6.3	-6.6	-6.6	-6.2	-6.4	-6.4	-6.8
90	-4.2	-4.0	-3.3	-4.0	-3.3	-3.3	-4.2
91	-5.2	-6.1	-5.9	-6.1	-5.4	-5.4	-6.3
101	-5.5	-7.5	-6.9	-5.8	-6.7	-6.7	-8.1
124	-6.0	-6.2	-5.3	-5.7	-5.4	-5.4	-6.6
168	-5.1	-5.2	-5.1	-5.0	-5.2	-5.2	-5.6
169	-4.8	-5.0	-4.1	-4.7	-4.6	-4.6	-4.3
177	-5.4	-5.1	-5.4	-5.2	-5.4	-5.4	-5.6
200	-6.0	-6.6	-6.7	-4.6	-5.2	-5.2	-6.9
245	-5.1	-2.5	-1.2	-1.8	-1.4	-1.4	-3.6
246	-5.3	-6.6	-7.2	-7.1	-7.5	-7.5	-6.8
253	-2.6	-2.3	-1.7	-4.6	-0.9	-0.9	-4.4
265	-4.8	-4.9	-4.3	-4.9	-4.8	-4.8	-4.3
278	-5.5	-5.7	-5.2	-5.7	-4.7	-4.7	-4.9
279	-4.7	-5.6	-4.9	-5.8	-4.9	-4.9	-5.4
289	-5.7	-5.8	-6.0	-6.0	-6.2	-6.2	-5.9
316	-5.7	-6.0	-5.7	-5.8	-5.9	-5.9	-5.8
360	-5.3	-4.9	-5.3	-5.0	-5.7	-5.7	-5.3
361	-5.7	-5.8	-5.6	-5.0	-5.7	-5.7	-5.9
369	-4.9	-5.0	-5.6	-5.7	-5.1	-5.1	-5.2
392	-4.9	-4.5	-2.9	-5.1	-3.6	-3.6	-4.9
437	-6.4	-6.7	-6.3	-6.3	-6.1	-6.1	-6.5
438	-6.3	-6.1	-6.0	-6.1	-6.0	-6.0	-6.1
448	-9.2	-9.1	-9.1	-9.2	-9.1	-9.1	-9.9
461	-1.3	-2.8	2.8	-2.4	-1.7	-1.7	-0.4
476	-6.5	-4.4	-5.8	-5.6	-6.0	-6.0	-5.1
477	-6.1	-5.8	-6.2	-6.0	-5.3	-5.3	-4.0
487	-6.3	-6.2	-5.8	-6.2	-6.2	-6.2	-6.0
514	-5.4	-5.2	-5.3	-5.3	-5.5	-5.5	-5.5
558	-3.4	-3.7	-3.5	-3.7	-3.7	-3.7	-3.6
559	-4.8	-4.4	-4.2	-4.4	-4.6	-4.6	-4.7
567	-0.6	-0.5	-0.6	-0.6	-0.6	-0.6	-0.6

Table SI11. Biological activities for BIN-T derivatives

	BIN-T	BIN-T-NH_2	BIN-T-NHR	BIN-T-OH	BIN-T-OR	BIN-T-NO_2	BIN-T-CN
Alpha-Radioprotector activity	0.9622	0.9805	0.9569	0.9768	0.7982	0.2875	0.1747
Gamma-radioprotector activity mechanism I	0.0001	0.0021	0.7028	0.0063	0.5842	0.6667	0.4270
Analgetic activity	0.9974	0.9970	0.9993	0.9962	0.9999	0.0000	0.8537
Anti-Adenovirus activity	0.6113	0.0508	0.0805	0.3068	0.5616	0.0000	0.0000
Anti-Arrhytmic activity	0.0000	0.0000	0.0000	0.0000	0.8874	0.1748	0.0051
Anti-Bacterial activity	0.0000	0.0000	0.0000	0.0000	0.0000	0.0000	0.0000
Anti-Oxidant activity	0.4819	0.4865	0.6316	0.4250	0.1175	0.8736	0.9080
Anti-Psychotic activity diazepine site	0.3933	0.6528	0.7869	0.5042	0.8201	0.9101	0.7382
Anti-Tumor Alkylic activity	0.6099	0.6181	0.7130	0.5870	0.5749	0.0482	0.1398
Anti-Tumor Antimitotic activity	0.8712	0.8477	0.9057	0.8245	0.9410	0.5231	0.6042
Anti-Tumor Cycline-dependent kinase 4 inhibitory activity	0.9586	0.9785	0.9887	0.9596	0.8466	0.1101	0.0331
Anti-Tumor Dihydrofolate reductase inhibitory activity	0.1093	0.1486	0.0723	0.0901	0.0070	0.0157	0.0135
Anti-Tumor DNA anti-metabolitic activity	0.5190	0.5402	0.5167	0.6178	0.0201	0.9847	0.9893
Anti-Tumor Topoisomerase I inhibitory activity	0.0000	0.0000	0.0000	0.0000	0.0064	0.0000	0.0000
Anti-Tumor Topoisomerase II inhibitory activity	0.9850	0.9900	0.9948	0.9835	0.9938	0.9144	0.9421
HIV1-proteaze inhibitory activity	0.3251	0.6082	0.3607	0.2577	0.8254	0.6909	0.7362
HT51 A inhibitory activity	0.4192	0.4592	0.0730	0.3149	0.3235	0.6063	0.1419
Tuberculostatic Dihydrofolate reductase inhibitory activity	0.8241	0.9082	0.8880	0.6865	0.9611	0.5479	0.8201
Human factor XA Inhibitory activity	0.0827	0.0000	0.0000	0.7353	0.0000	0.8475	0.9976
Metabolism at CYP450 2D6	0.8239	0.9234	0.4072	0.9172	0.7863	0.9525	0.9534
Metabolism at CYP450 3A4	0.9796	0.9835	0.7947	0.9772	0.9884	0.9897	0.9971
Progestagenic activity	0.9970	0.9990	0.9979	0.9976	0.9998	0.0000	0.0006
Vasorelaxant_activity	0.0019	0.0000	0.0000	0.0062	0.0000	0.7761	0.6993
COX1 inhibitory activity	0.9489	0.9253	0.9128	0.9624	0.7266	0.6541	0.8284
COX2 inhibitory activity	0.0066	0.1151	0.5764	0.0376	0.0000	0.0000	0.0005
LOX inhibitory activity	0.9674	0.9226	0.9638	0.9550	0.8777	0.0000	0.0000

Table SI12. Biological activities for BIN derivatives

	BIN	BIN-NH_2	BIN-NHR	BIN-OH	BIN-OR	BIN-NO_2	BIN-CN
Alpha-Radioprotector activity	0.8196	0.9570	0.8385	0.8902	0.9003	0.5686	0.8684
Gamma-radioprotector activity mechanism I	0.5000	0.6080	0.2073	0.0106	0.0439	0.0022	0.0100
Analgetic activity	0.0991	0.2240	0.9666	0.0000	0.9531	0.0000	0.0001
Anti-Adenovirus activity	0.0000	0.0009	0.7220	0.1322	0.9447	0.2521	0.2843
Anti-Arrhytmic activity	0.2960	0.0022	0.9703	0.5925	0.7894	0.9482	0.7626
Anti-Bacterial activity	0.1914	0.0656	0.5679	0.7965	0.1850	0.7994	0.8052
Anti-Oxidant activity	0.0000	0.0000	0.2715	0.0000	0.3995	0.0407	0.0000
Anti-Psychotic activity diazepine site	0.4555	0.3506	0.8854	0.1307	0.2691	0.2770	0.1110
Anti-Tumor Alkylic activity	0.4472	0.3561	0.5869	0.3447	0.5255	0.3652	0.3728
Anti-Tumor Antimitotic activity	0.1436	0.1110	0.0471	0.1279	0.0528	0.1618	0.1159
Anti-Tumor Cycline-dependent kinase 4 inhibitory activity	0.7125	0.9176	0.0000	0.5818	0.0007	0.6316	0.4891
Anti-Tumor Dihydrofolate reductase inhibitory activity	0.0511	0.1729	0.2079	0.0512	0.1723	0.0993	0.0693
Anti-Tumor DNA anti-metabolitic activity	0.9093	0.8256	0.3907	0.8666	0.2542	0.8949	0.9157
Anti-Tumor Topoisomerase I inhibitory activity	0.9843	0.6425	0.0000	0.9991	0.0000	0.9974	0.9993
Anti-Tumor Topoisomerase II inhibitory activity	0.3537	0.8739	0.8230	0.6246	0.7861	0.7291	0.6574
HIV1-proteaze inhibitory activity	0.2974	0.4583	0.5622	0.0902	0.2973	0.1340	0.0912
HT51 A inhibitory activity	0.2189	0.3627	0.0000	0.0012	0.0000	0.0000	0.0000
Tuberculostatic Dihydrofolate reductase inhibitory activity	0.9912	0.9971	0.8944	0.5083	0.9785	0.7790	0.5400
Human factor XA Inhibitory activity	0.9926	0.9434	0.9703	0.9999	0.9997	0.5847	0.9930
Metabolism at CYP450 2D6	0.8812	0.8614	0.5392	0.6337	0.9112	0.7537	0.6168
Metabolism at CYP450 3A4	0.9979	0.9979	0.9988	0.8639	0.9996	0.9196	0.8760
Progestagenic activity	0.9679	0.8797	0.3993	0.6726	0.0264	0.9879	0.9493
Vasorelaxant_activity	0.1269	0.0177	0.0037	0.2859	0.1383	0.2555	0.0940
COX1 inhibitory activity	0.6328	0.0203	0.0000	0.0001	0.0000	0.0000	0.0000
COX2 inhibitory activity	0.9999	0.7328	0.9995	1.0000	0.9978	0.9999	1.0000
LOX inhibitory activity	0.0003	0.0062	0.0463	0.0000	0.0281	0.0000	0.0000
